# MiR-508-3p promotes proliferation and inhibits apoptosis of middle ear cholesteatoma cells by targeting PTEN/PI3K/AKT pathway

**DOI:** 10.7150/ijms.60907

**Published:** 2021-07-11

**Authors:** Dongliang Liu, Xiulan Ma

**Affiliations:** Department of Otolaryngology Head and Neck Surgery, Shengjing Hospital of China Medical University, Liaoning 110004, China

**Keywords:** hsa_circ_0000007, miR-508-3p, PTEN/ PI3K/Akt pathway, cholesteatoma, proliferation and apoptosis

## Abstract

Cholesteatoma of the middle ear is a common disease in otolaryngology, which can lead to serious intracranial and extracranial complications. Recent studies showed that the dysregulation of microRNA may be involved in the formation of middle ear cholesteatoma. This study aimed to explore the regulatory effect of micro ribonucleic acid 508-3p (*miR-508-3p*) on proliferation and apoptosis of middle ear cholesteatoma cells and excavate its underlying regulatory mechanism. We found *miR-508-3p* expression was upregulated in tissues and cells of cholesteatoma which was inversely related to the expression of *hsa_circ_0000007.* Overexpression of *miR-508-3p* could notably facilitate cholesteatoma cell proliferation. Luciferase reporter assay showed that *miR-508-3p* bound the 3'-untranslated region of its downstream mRNA *PTEN*. Gain and loss of functions of *miR-508-3p* were performed to identify their roles in the biological behaviors of cholesteatoma cells, including proliferation and apoptosis. Rescue assays confirmed that PTEN could reverse the effect of *miR-508-3p* overexpression on cell proliferation. In a word, this study validated that the development of cholesteatoma may regulated by *hsa_circ_0000007*/*miR-508-3p*/ PTEN/ PI3K/Akt axis.

## Introduction

Cholesteatoma is benign collections of keratinized squamous epithelium within the middle ear. There are congenital and acquired middle ear cholesteatomas 1. Congenital cholesteatoma is formed from remnants of epithelium that get trapped in the temporal bone during development 2. Acquired cholesteatoma does not result from an embryologic phenomenon, but are the result of pathologic changes that cause the uncontrolled growth of squamous keratinized epithelium in the middle ear 3. This study is aimed at the acquired cholesteatoma. When the cholesteatoma begins, it can damage temporal bone and nearby structures like ossicles, facial nerve, vestibule, semicircular canal and brain causing many problems like hearing loss, facial paralysis, dizziness, encephalopyosis and so on. Cholesteatoma can be a difficult disease to treat because the underlying cause of the disease, eustachian tube dysfunction, is generally not addressed. This can lead to recurrent disease. Surgical resection of cholesteatoma can also be quite challenging, and residual cholesteatoma is often present after surgery 4. The pathogenesis of acquired cholesteatoma is not clear. The most popular theory is the keratinocyte of the middle ear becomes hyperproliferative.

Circular RNA (circRNA) was considered as a class of endogenous noncoding RNA (ncRNA) 5. CircRNA is mainly located in the cytoplasm and is highly stable compared to other ncRNAs 6. CircRNA is abundantly expressed and evolutionarily conserved across eukaryotic organisms 7 and it plays crucial roles in many diseases, including digestive system neoplasms, cardiovascular disease, and Osteosarcoma 8-10. It was commonly known that circRNAs regulated cell functions and cancer development by sponging microRNAs (miRNAs) 11-13.

MicroRNAs (miRNAs) are small endogenous RNAs that regulate gene expression post-transcriptionally. MiRNAs are short non-coding RNAs of 19~25 nucleotides that mediate gene silencing by guiding Argonaute (AGO) proteins to target sites in the 3' untranslated region (UTR) of mRNAs. AGOs constitute a large family of proteins that use single-stranded small nucleic acids as guides to complementary sequences in RNA or DNA targeted for silencing 14. The miRNA-loaded AGO forms the targeting module of the miRNA-induced silencing complex (miRISC), which promotes translation repression and degradation of targeted mRNAs 15. A single miRNA can target hundreds of mRNAs and influence the expression of many genes often involved in a functional interacting pathway 16.

The PTEN/PI3K/AKT pathway regulates multiple cellular functions, including cell growth, differentiation, proliferation, survival, motility, invasion and intracellular trafficking in various diseases like lung cancer, gastric cancer, breast cancer and so on 17-19. PTEN, a dual protein and lipid phosphatase, primarily dephosphorylates phosphatidylinositol-3,4,5-trisphosphate (PIP3), which is the product of PI3K and is able to recruit Akt to the membrane, where it is phosphorylated and stimulated 20. Activated Akt may regulate multiple biological processes, including cell survival, metabolism, cell proliferation and growth, by affecting its downstream substrates 21,22.

Taken together, the current study was designed to explore the role of *hsa_circ_0000007* and *miR-508-3p* in the development of cholesteatoma with the involvement of the PTEN/PI3K/AKT signaling pathway.

## Materials and Methods

### Patients and samples

The present study was performed using data obtained randomly from 20 patients. All patients were surgically treated at Shengjing Hospital of China Medical University from September 1, 2020 to December 31, 2020. All patients have received pathological diagnosis of middle ear cholesteatoma. We collected and frozen all samples. The lower age limit of these patients was 18 years, and the higher limit was 70 years, with the median age of 53.57±18.67 years, which included 7 women and 13 men. At the same time, 15 cases of posterior auricular normal skin or skin fragments that could not be used during otoplasty were collected as control group. This study was approved by the Institutional Human Ethics Committee of Shengjing Hospital of China Medical University, and prior informed consent obtained from all the patients.

### Data source

The microarray data analyzed in this study were obtained from the Gene Expression Omnibus (GEO) (https://www.ncbi.nlm.nih.gov/geo/), accession number GSE102715, published on Apr 27, 2020. GEO is a public functional genomics data repository supporting MIAME-compliant data submissions. Array- and sequence-based data are accepted. Tools are provided to help users query and download experiments and curated gene expression profiles 23,24. This dataset GSE102715 profiled the differences in circRNA expression between 4 pairs of cholesteatoma (GSM2743683, GSM2743685, GSM2743687, GSM2743689) and matched normal skin samples (GSM2743684, GSM2743686, GSM2743688, GSM2743690). All specimens were obtained from 2 female and 2 male patients aged 18-year-old to 32-year-old who received unilateral middle ear cholesteatoma surgeries. The post-auricular skins were taken as control samples from the same patients. GSE102715 was based on the Agilent GPL21825 platform (Arraystar Human CircRNA microarray V2). All of the data were freely available online.

### Data processing and differential expression analysis

After getting raw expression data, the volcano figure was created using GraphPad Prism 7.0 software. Differentially expressed genes (DEGs) analysis between cholesteatoma and normal samples was performed using the online analysis tool GEO2R (www.ncbi.nlm.nih.gov/geo/geo2r/?acc=GSE102715) and NetworkAnalyst 3.0 (www.networkanalyst.ca/NetworkAnalyst/home.xhtml). The intersecting part between the two analysis was identified using the Venn diagram webtool (http://bioinformatics.psb.ugent.be/webtools/Venn/). The adjusted *P* value and |logFC| were calculated. Genes that met the cutoff criteria, adjusted *P* value<0.05 and |logFC|≥2.0, were considered as DEGs. The heatmap for the DEGs was created using GraphPad Prism 7.0 software.

### Functional enrichment analysis

GO analysis is a common useful method for large scale functional enrichment research; The GO analysis included 3 categories, namely, biological process (BP), cellular component (CC) and molecular function (MF), which were used to predict protein functions 25. Pathway functional analysis was performed on the Kyoto Encyclopedia of Genes and Genomes (KEGG) database 26. GO annotation analysis and KEGG pathway enrichment analysis of DEGs in this study was performed using the clusterProfiler of Limma R package available on Bioconductor (http://bioconductor.org/packages/release/bioc/html/limma.html). P<0.05 and gene counts≥2 were considered statistically significant.

### Prediction of circRNA-miRNA-mRNA target gene associations

We predicted circRNA/miRNA target genes using online tools Circular RNA Interactome (https://circinteractome.irp.nia.nih.gov/mirna_target_sites.html), and predicted the interactive relationships between miRNA and target mRNA using TargetScan Human 7.2 (http://www.targetscan.org/vert_72/).

### Cell culture and transfection

After extraction of cholesteatoma tissues from middle ear, the tissues were washed 3 times with pre-cooled phosphate buffer solution (PBS) and cut into 1×1 mm^3^ blocks using a surgical scissor with high temperature sterilization. Digestion of them with 0.25% pancreatin at 37℃ for 3h after centrifugation was terminated by adding culture medium. The filtrate filtered by a 200mesh cell sieve was collected and centrifuged at 1500 rpm for 10 min, then the supernatant was discarded, and the complete medium was added to re-suspend cell precipitate for subsequent experiments. The passage cells were re-inoculated in a 6-well plate. After the cell fusion degree reached about 80%, *miR-508-3p* NC, *miR-508-3p* mimic and *miR-508-3p* inhibitor GenePharma (Shanghai, China) were transfected into middle ear cholesteatoma cells respectively according to the instructions of Lipofectamine^TM^2000 (Invitrogen, Carlsbad, CA, USA) transfection reagent, and cultured in a CO_2_ incubator at 37°C. After 48 hours, ELISA, EdU staining and TUNEL staining were explored, and the transfection efficiency was evaluated using RT-qPCR. Experimental line of human immortalized keratinocytes (HaCaT) was obtained from shanghai Zhong Qiao Xin Zhou Biotechnology Co.,Ltd. Cells were cultured in high-glucose Dulbecco's Modified Eagle Media (DMEM) (HyClone, Thermo Fisher, Shanghai, China) with 10% fetal bovine serum (FBS) (Corning, Thermo Fisher, Waltham, MA). HaCaT cells were grown under sterile, humidified conditions at 37℃ and 5% CO_2_.

### RT-qPCR

Total RNAs were separated by using trizol reagent (Takara, Otsu, Japan). And Prime Script RT Reagent Kit (HaoranBio, Xuhui, Shanghai, China) and TaqMan^TM^ Advanced miRNA cDNA Synthesis Kit (Waltham, MA, USA) were then respectively applied to synthesize complementary DNA. Subsequently, the SYBR Green Master Mix (Takara, Dalian, China) was utilized to conduct the RT-qPCR on ABI 7500 System (Applied iosystems, Carlsbad, California). GAPDH and U6 served as internal controls. Relative expression of RNAs was calculated by using the 2^-ΔΔCt^ method.

### CCK-8

Cell counting kit-8 (CCK-8) reagent (Beyotime Institute of Biotechnology, Shanghai, China) was used to perform CCK-8 assay in accordance with the manufacturer's suggestions. Transfected cells (1 × 10^3^) were seeded into the 96-well plates and cultured for 0, 24, 48, 72 and 96 h. Then each well was added with CCK-8 reagent. After 4 h incubation, the optical density was measured using a microplate reader at a wavelength of 450 nm to detect cell proliferation at each time points. The detection was repeated 3 times.

### ELISA

The ELISA kit was taken out from a low-temperature refrigerator, and left at room temperature, and then standard substances and diluents were added into blank holes according to instructions of the ELISA kit, and standard substances with different concentrations were added into the rest holes to draw standard curves. Then diluted enzyme conjugate was added, incubated at 37℃ for 30 min and washed for 5 times, and 100μL of chromogenic substrate was added, and then incubated in dark for 15 min. Finally, the reaction termination solution was added dropwise, and absorbance was detected with a microplate reader.

### 5-ethynyl-2'-deoxyuridine (EdU) staining

Each well was added with 100μL of penetrant, incubated for 15 min, washed with PBS, then added with 100μL of EdU staining solution diluted with culture medium, and incubated for 30 min. The culture medium was discarded and the cells were decolorized with PBS for 3 times, each time for 5 min. The staining was observed under a fluorescence microscope.

### TUNEL staining

Cells in each group were washed with PBS, fixed with 4% paraformaldehyde for 30 min, permeabilized with 0.3% TritonX-100 solution for 15 min, added with 50μL of TUNEL test solution per well, incubated at 37℃ in dark for 60 min, washed with PBS, added with anti-fluorescence quencher, and observed via TUNEL staining under a microscope.

### Western blot

The cells from each group were collected and lysed with RIPA lysis buffer, and the protein concentrations in each group were determined by Braford method. At room temperature, proteins were separated by 10% SDS-PAGE at constant pressure, transferred to a PVDF membrane, and sealed with 5% skimmed milk powder for 1 h. After that, the membrane was incubated with primary anti-antibodies PTEN (1:1000), PI3K (1:1000) and p-Akt (1:1000) overnight at 4℃ and then the HRP labeled secondary antibody (1: 2000) for 2 h. The bands were developed using DAB color development method, and the absorbance of each band was analyzed by Image J software.

### Luciferase reporter assay

Reporter plasmids were obtained by inserting *PTEN* 3'-UTR sequence into pmirGLO vector (Promega, Madison, WI, USA). For the luciferase assay, *miR-508-3p* mimics and reporter plasmids were co-transfected into 239T cells using Lipofectamine^2000^. After culturing for 48 h, firefly and Renilla luciferase activities were measured using the Dual Luciferase Reporter Assay System (Promega, Sunnyvale, CA, USA) according to the manufacturer's instructions.

### Statistical analysis

The statistical analysis was performed using the SPSS 20.0 and the data were visualized using the GraphPad 7. Data has been displayed as the mean± standard deviation (SD). The one-way ANOVA or student's t-test was utilized for the comparisons among groups. Pearson analysis was used to observe the correlation between *hsa_circ_0000007* and *miR-508-3p* in tissue samples. Each experiment of this study was performed in triplicate. Any value of *p* < 0.05 was thought to be of statistical significance.

## Results

### Identification of different expression circRNA

**FIGURE 1:**
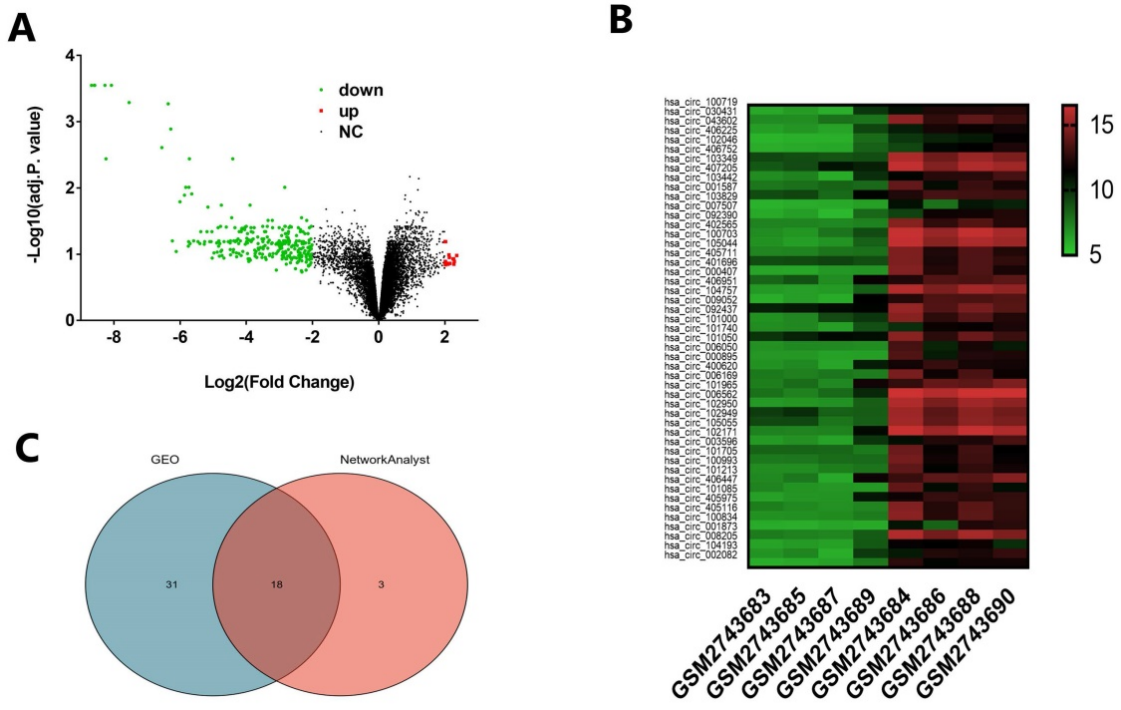
** Distribution of differentially expressed circRNAs between cholesteatoma and normal skin.** (A) Volcano plot representing log_2_(fold change) and -log10 (adjusted. p. value). Red stands for upregulations, green stands for downregulations and black stands for normal expression in volcanoes. Each point represents a gene. (B) Heatmap of differentially expressed circRNAs in cholesteatoma and normal skin with the criteria |log_2_FoldChange|>2 and adjusted p. value < 0.05. The horizontal axis represents the names of the samples. GSM2743683, GSM2743685, GSM2743687and GSM2743689 are the samples of cholesteatoma. GSM2743684, GSM2743686, GSM2743688 and GSM2743690 represent normal skin. The vertical axis represents differentially expressed circRNAs. Similar with Volcano, red stands for upregulations, green stands for downregulations and black stands for normal expression. (C) Venn diagram showing the intersection of significative dysregulated circRNA between GEO2R and NetworkAnalyst 3.0.

We downloaded the microarray expression dataset GSE102715 from Gene Expression Omnibus (GEO) and analyzed the different circRNAs between cholesteatoma and normal skin using the online analysis tool GEO2R and NetworkAnalyst 3.0. In total, there are 13247 raw circRNAs in dataset GSE102715. Based on the criteria of |logFC|≥2, GEO2R identified 16 upregulated and 283 downregulated circRNAs showed in Volcano (Fig. 1A). Based on the criteria of adjusted *p*. value <0.05 and |logFC|≥2, We obtained top 49 different circRNAs to show in heatmap (Fig. 1B). NetworkAnalyst 3.0 identified 21 dysregulated circRNAs on the criteria of* P*<0.05 and |logFC|≥2. Subsequently, Venn analysis was performed to get the intersection of the dysregulated circRNAs between GEO2R and NetworkAnalyst 3.0 result (Fig. 1C). As showed in Venn diagram, there are 18 common candidates of dysregulated circRNAs.

### Functional enrichment analysis of DEGs

**FIGURE 2:**
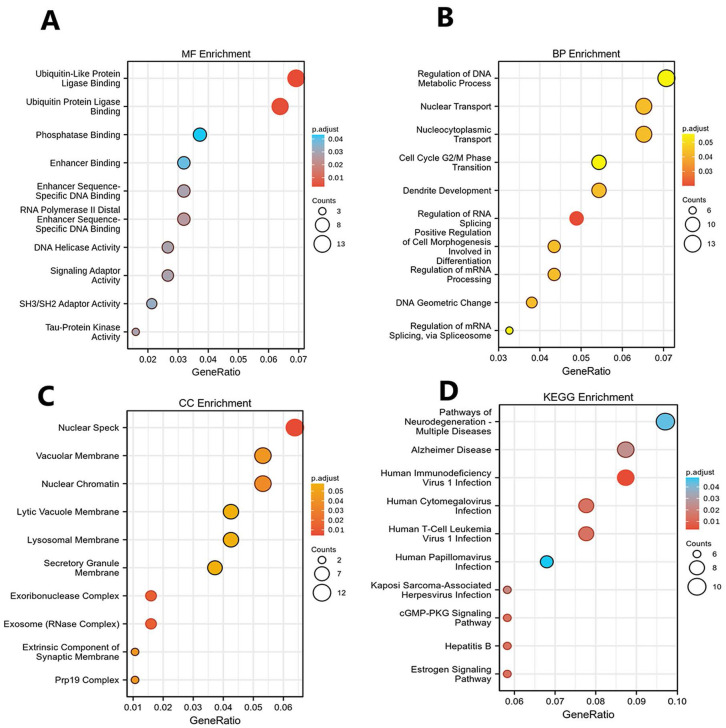
** GO and KEGG analysis.** (A) Top 10 significant biological process (BP) GO functional terms; (B) Top 10 significant cellular component (CC) GO functional terms; (C) Top 10 significant molecular function (MF) GO functional terms in cholesteatoma compared to the normal skin, respectively. (D) Top 10 enriched pathways identified by KEGG analysis. The X-axis represents the gene ratio for each of the differentially expressed genes in each pathway. The Y-axis gives the name of the enriched term. The size of each node indicates the number of significant genes in each category. The adjust p value are indicated by changing colors.

GO function and KEGG pathway enrichment analysis for DEGs were showed in (Figs. 2A-D). The enriched GO terms were divided into CC, BP, and MF ontologies. The results of GO analysis indicated that DEGs were mainly enriched in BPs, including regulation of DNA metabolic process (GO:0051052), nucleocytoplasmic transport (GO:0006913), nuclear transport (GO:0051169), regulation of chromosome organization (GO:0033044), ncRNA processing (GO:0034470) and so on. MF analysis showed that the DEGs were significantly enriched in ubiquitin-like protein ligase binding (GO:0044389), ubiquitin protein ligase binding (GO:0031625), protein serine/threonine kinase activity (GO:0004674), catalytic activity, acting on RNA (GO:0140098), phospholipid binding (GO:0005543) and so on. For the cell component, the DEGs were enriched in nuclear speck (GO:0016607), nuclear chromatin (GO:0000790), vacuolar membrane (GO:0005774), lysosomal membrane (GO:0005765), lytic vacuole membrane (GO:0098852) and so on. In addition, KEGG pathway analysis (Fig. 2D) showed that upregulated genes were mainly enriched in Pathways of neurodegeneration - multiple diseases (KEGG ID: hsa05022), Human immunodeficiency virus 1 infection (KEGG ID: hsa05170), Alzheimer disease (KEGG ID: hsa05010), Human T-cell leukemia virus 1 infection (KEGG ID: hsa05166), Human cytomegalovirus infection (KEGG ID: hsa05163) and so on. The top ten of *P* < 0.05 were chosen in each enrichment.

### Identification of genes of interest

**FIGURE 3:**
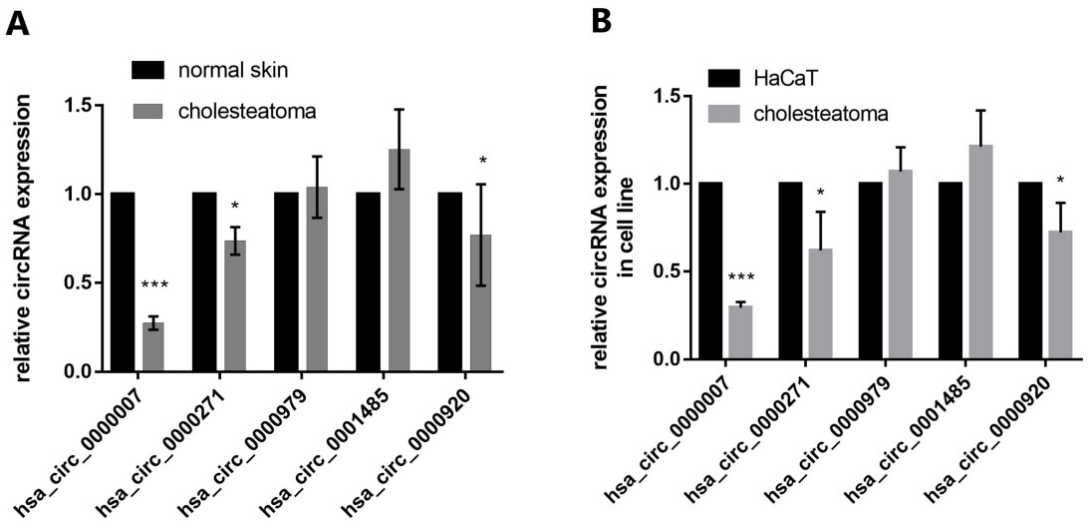
** The expression of 5 candidate circRNAs in both specimens and cell lines.** (A) RT-qPCR detection show the expression of 5 candidate circRNAs (*hsa_circ_0000007, hsa_circ_0000271, hsa_circ_0000979, hsa_circ_0001485, hsa_circ_0000920*) in both cholesteatoma (*n*=10) and normal skin specimens (*n*=10). (B) RT-qPCR assay was conducted to detect the expression of the 5 candidate circRNAs in cholesteatoma cells and HaCaT cell line. Data are presented as mean ± SD. *p < 0.05, **p < 0.01. ***p < 0.001. Repetition=3

We identified five candidates of circRNAs (*hsa_circ_0000007*, *hsa_circ_0000271, hsa_circ_0000979, hsa_circ_0001485, hsa_circ_0000920*) which are markedly downregulated in cholesteatoma to be further studied. RT-qPCR assay depicted that *hsa_circ_0000007* expression was most significantly downregulated in comparison with other 4 circRNAs both in tissue and cells (Fig. 3A and B).

### Prediction and verification of potential target microRNA and mRNA

**FIGURE 4:**
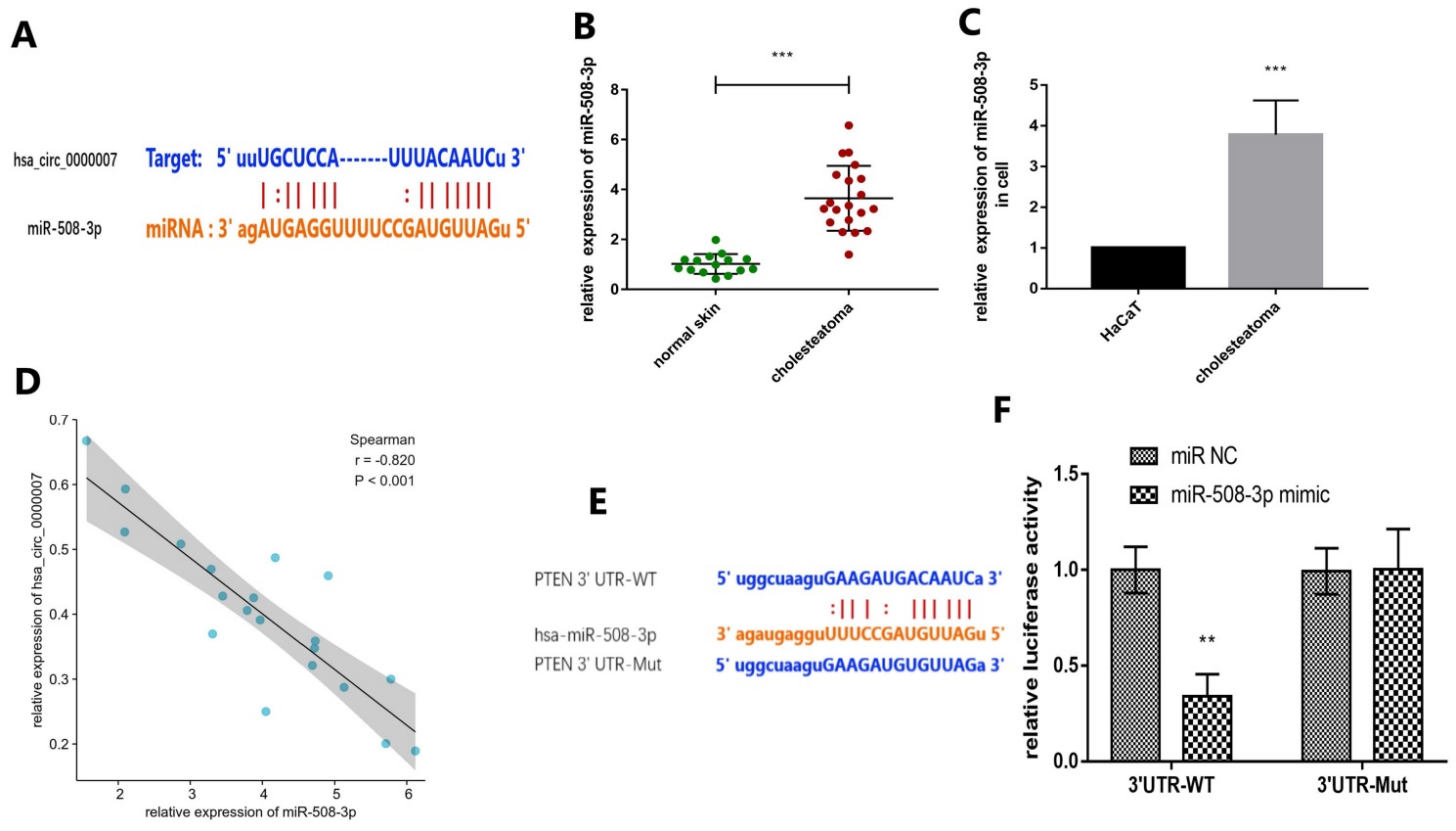
** Prediction and verification of potential target miRNA and mRNA.** (A) Prediction of the binding of *miR-508-3p* and *hsa_circ_0000007* performed via an online website (https://circinteractome.irp.nia.nih.gov/). (B)* MiR-508-3p* levels in 20 cholesteatoma tissues and 15 normal skin tissues were measured by RT-qPCR. (C) Expression of *miR-508-3p* in cholesteatoma cells and HaCaT cell line were measured by RT-qPCR. (D) The negative correlation between the expressions of *miR-508-3p* and *hsa_circ_0000007* was verified by Spearman test (r = -0.820, *p*< 0.001). (E) Prediction of the binding of *miR-508-3p* and *PTEN* performed by an online website (http://www.targetscan.org). (F) Binding relation of *miR-508-3p* and *PTEN* identified using dual luciferase reporter gene assay. *p < 0.05, **p < 0.01. ***p < 0.001.

We intended to explore the molecular mechanism of *hsa_circ_0000007* in cholesteatoma. We used online prediction tool Circular RNA Interactome to predict potential miRNA which could possibly bind with *hsa_circ_0000007*. The prediction results showed four miRNAs, including *miR-492, miR-508-3p, miR-665 and miR-876-3p*. We chose *miR-508-3p* because its context+ score was minimum of all (Fig. 4A). In this study, RT-qPCR assay was applied to examine the expression of *miR-508-3p* both in cholesteatoma tissue and cells (Fig. 4B and C). The results demonstrated that the expression of *miR-508-3p* was notably higher in cholesteatoma tissue and cells than that in normal skin and HaCaT cells (*p*<0.001). Moreover, *miR-508-3p* expression was negatively correlated with *hsa_circ_0000007* expression (Fig. 4D) (*p*<0.001). Next, bioinformatics analysis tool Targetscan (http://www.targetscan.org/) showed that *PTEN* was a potential *miR-508-3p* target mRNA. *PTEN* was also found to have a binding site for *miR-508-3p* through searching starBase (Fig. 4E). To confirm that *PTEN* was a *miR-508-3p* target, we cloned mutant and wild-type* PTEN* sequences to construct mutant vectors and reporter plasmids respectively. The results showed that the reporter plasmid and *miR-508-3p* mimic co-transfections visibly suppressed luciferase activity and mutated *PTEN* vectors, but *miR-508-3p* mimic co-transfection had no significant effect on luciferase activity. These results proved that* miR-508-3p* directly targeted* PTEN* (Fig. 4F).

### Effect of *miR-508-3p* on the biological phenotype of cholesteatoma cells

**FIGURE 5:**
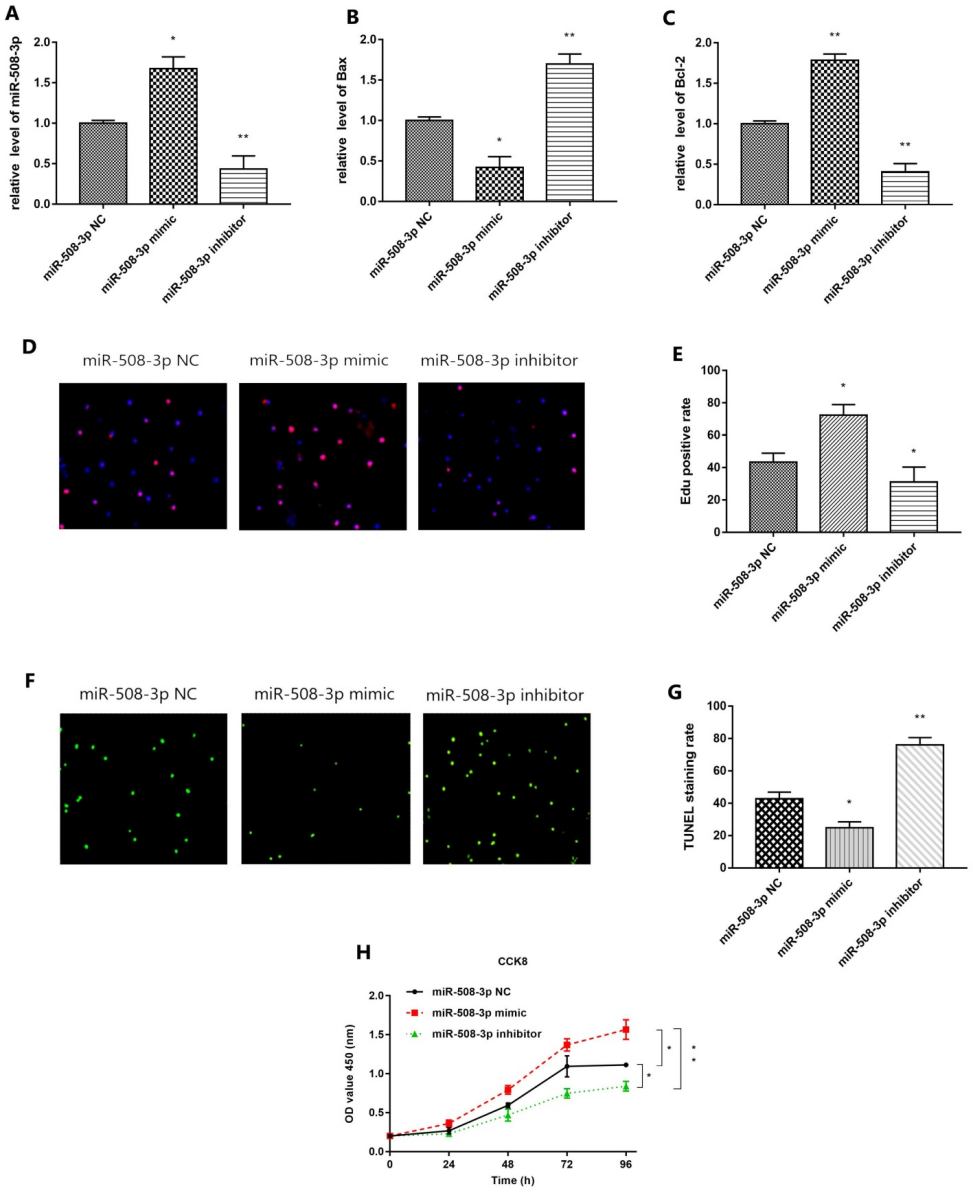
** Effect of *miR-508-3p* on proliferation and apoptosis in cholesteatoma.** (A) RT-qPCR detection show the expression of *miR-508-3p* in *miR-508-3p* NC group, *miR-508-3p* mimic group and *miR-508-3p* inhibitor group. (B, C) Level of Bax and Bcl-2 in *miR-508-3p* NC group, *miR-508-3p* mimic group and *miR-508-3p* inhibitor group of cholesteatoma cells via ELISA. (D) EdU staining of transfected cells. (E) EdU positive rate of* miR-508-3p* NC group, *miR-508-3p* mimic group and *miR-508-3p* inhibitor group. (F) TUNEL staining of transfected cells. (G) TUNEL staining rate of *miR-508-3p* NC group, *miR-508-3p* mimic group and *miR-508-3p* inhibitor group. (H) The proliferative capability of transfected cells was evaluated by CCK-8 assay. *p < 0.05, **p < 0.01. ***p < 0.001

QRT-PCR results are shown in Fig. 5A. Compared with that in *miR-508-3p* NC group, *miR-508-3p* level in middle ear cholesteatoma cells overtly increased in *miR-508-3p* mimic group (*p*<0.05) and notably decreased in *miR-508-3p* inhibitor group (*p*<0.01). The results of ELISA are shown in Fig. 5B and 5C. Compared with those in *miR-508-3p* NC group, Bax level in middle ear cholesteatoma cells was decreased in *miR-508-3p* mimic group(*p*<0.05) and enhanced in *miR-508-3p* inhibitor group (*p*<0.01) respectively, while Bcl-2 level was elevated in *miR-508-3p* mimic group (*p*<0.01) and declined in *miR-508-3p* inhibitor group (*p*<0.01) respectively. Fig. 5D presents the results of EdU staining. Compared with that in *miR-508-3p* NC group, the proliferation rate of middle ear cholesteatoma cells was increased in *miR-508-3p* mimic group(*p*<0.05) and decreased in *miR-508-3p* inhibitor group (*p*<0.05), as shown in Fig. 5E. Fig. 5F shows TUNEL staining results. Compared with that in *miR-508-3p* NC group, the apoptosis rate of middle ear cholesteatoma cells was lowered in *miR-508-3p* mimic group (*p*<0.05) and elevated in *miR-508-3p* inhibitor group (*p*<0.01), as shown in Fig. 5G. The CCK-8 assay results suggested that over-expression of *miR-508-3p* significantly promoted the cholesteatoma cell proliferation 96 h after transfection, while down-expression of *miR-508-3p* reduced proliferation (all *p*< 0.05) (Fig. 5H). In conclusion, *miR-508-3p* can promote proliferation and inhibit apoptosis in cholesteatoma cells.

### *MiR-508-3p* facilitates cell proliferation and inhibits apoptosis in cholesteatoma cell through PTEN/PI3K/Akt signal pathway

**FIGURE 6:**
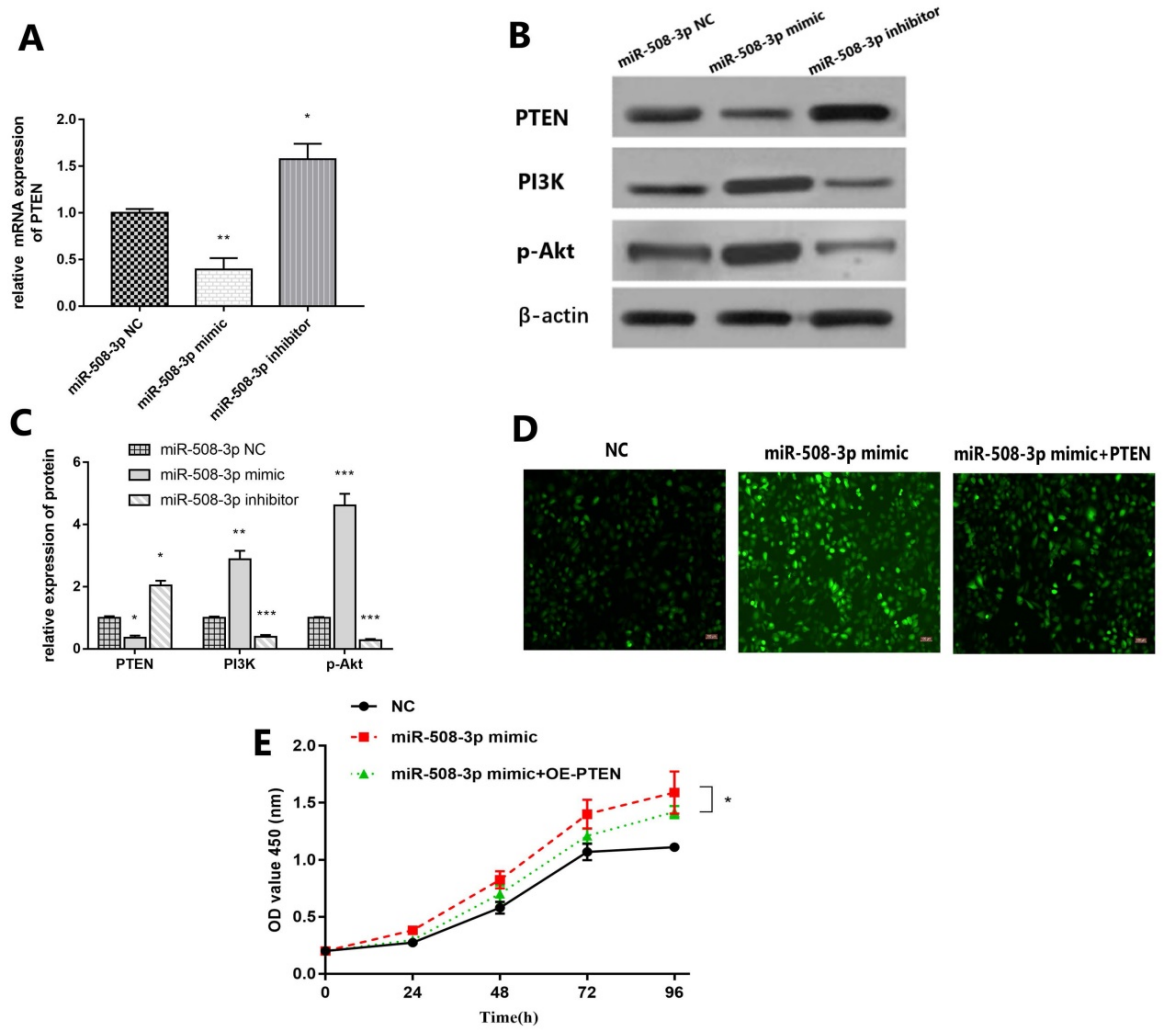
** Upregulation of PTEN reverses the effect of *miR-508-3p* on cholesteatoma.** (A) The expression of *PTEN* was detected by RT-qPCR in *miR-508-3p* NC group, *miR-508-3p* mimic group and *miR-508-3p* inhibitor group. (B) Western blot analysis showed the protein level of PTEN, PI3K and p-Akt in *miR-508-3p* NC group, *miR-508-3p* mimic group and *miR-508-3p* inhibitor group in vitro. (C) Relative protein level of PTEN, PI3K and p-Akt in vitro. (D) The transfected cells growth situation of *miR-508-3p* NC, *miR-508-3p* mimic and *miR-508-3p+PTEN*. (E) The proliferative capability of transfected cells was evaluated by CCK-8 assay. *p < 0.05, **p < 0.01. ***p < 0.001

QRT-PCR results are shown in Fig. 6A. Compared with that in *miR-508-3p* NC group, *PTEN* level in middle ear cholesteatoma cells notably decreased in *miR-508-3p* mimic group (*p*<0.01) and obviously increased in *miR-508-3p* inhibitor group (*p*<0.05). The Western blotting results are revealed in Fig. 6B and 6C. Compared with those in *miR-508-3p* NC group, the level of PTEN protein in middle ear cholesteatoma cells decreased in *miR-508-3p* mimic group and increased in *miR-508-3p* inhibitor group (*p*<0.05), while the levels of PI3K and p-Akt proteins raised in *miR-508-3p* mimic group (*p*<0.01, *p*<0.001) and lowered in *miR-508-3p* inhibitor group (*p*<0.001, *p*<0.001). Fig. 6D and 6E suggested that over-expression of *miR-508-3p* visibly facilitated the cholesteatoma cell proliferation after transfection. Co-transfection with *miR-508-3p* mimic and oe-*PTEN* led to significantly reduced cell proliferation compared to *miR-508-3p* mimic transfection alone (*p* < 0.05). This rescue assays were performed to prove overexpression *PTEN* can reverse the trendy of proliferation after upregulation of *miR-508-3p*.

## Discussion

In recent years, with the gradual maturity of biochip and sequencing technology, a variety of biological databases can provide more reliable data for researchers 23,27. Subsequently, Non-coding RNA (ncRNA) has been increasingly studied in various diseases 28. Studies have shown that ncRNAs play important roles in various biological processes 29,30. NcRNAs are commonly employed for RNA that does not encode a protein, but can regulate biological transcription and translation. NcRNAs include miRNAs, circRNAs and so on 31.

MicroRNAs (miRNAs), widely distributed, small regulatory RNA genes, target both messenger RNA (mRNA) degradation and suppression of protein translation based on sequence complementarity between the miRNA and its targeted mRNA 32. MiRNAs are involved in human health and disease as endogenous suppressors of the translation of coding genes. Specific cognate mRNA targets for miRNA are the key to the regulation of mRNA 33. MiRNAs have been reported and studied in various diseases. For instance, microRNA is a potential blood-based epigenetic biomarker for Alzheimer's disease 34. Epigenetic abnormalities in meningiomas include abnormal microRNA expression 35. Many studies have found that miRNA is most closely related to proliferation and apoptosis. For example, Xia MM et al. 36 summarized many microRNAs can regulate Sertoli cell proliferation and adhesion. Zhu ZJ et al. 37 found overexpression of microRNA-181a (miR-181a) promoted the proliferation and inhibited the apoptosis of osteosarcoma cells.

Circular RNAs (circRNAs), a novel class of long noncoding RNAs, are characterized by a covalently closed continuous loop without 5′ or 3′ polarities structure and have been widely found in thousands of lives including plants, animals and human beings. Utilizing the high-throughput RNA sequencing (RNA-seq) technology, recent findings have indicated that a great deal of circRNAs, exhibit cell type-specific, tissue-specific or developmental-stage-specific expression. Evidences are arising that some circRNAs might regulate microRNA (miRNA) function as microRNA sponges and play a significant role in transcriptional control. CircRNAs associate with related miRNAs and the circRNA-miRNA axes are involved in a serious of disease pathways such as apoptosis, vascularization, invasion and metastasis 38. The aberrant expression of circRNAs has been reported in many human diseases including gastric cancer 39, colorectal cancer 40, papillary thyroid cancer 41, lung adenocarcinoma 42 and so on. Function of circRNA-miRNAs-mRNA axis is increasingly studied in human diseases, but has not been reported in middle ear cholesteatoma. In this experiment, we carried out a study on *hsa_circ_0000007*-*miR-508-3p*-*PTEN* axis.

Cholesteatoma is a noncancerous cystic lesion derived from an abnormal growth of keratinizing squamous epithelium in the temporal bone 43. Although not malignant, cholesteatoma can destroy temporal bone and nearby structures like ossicles, facial nerve, vestibule, semicircular canal and brain causing many problems like hearing loss, facial paralysis, dizziness, encephalopyosis and so on. Cholesteatoma is a serious disease in otolaryngology. Most of the cholesteatoma mechanisms that have been proposed to explain the pathogenesis of acquired cholesteatoma can be divided into four categories: (1) invagination theory (retraction pocket theory), (2) the theory of epithelial invasion or migration (immigration theory), (3) the theory of squamous metaplasia, and (4) basal cell hyperplasia theory (papillary ingrowth theory) 44. Ultimately, the accumulation of epithelial keratinocytes with over-proliferation and inhibited apoptosis in a deepening retraction pocket leads to the formation of cholesteatoma.

With the increasing research on ncRNA in recent years, many RNA microarray databases 45 can be used for free. In this study, we mined GSE102715 in GEO database. By obtaining the raw data, we found *hsa_circ_0000007* through two analysis software (GEO2R and Network Analyst 3.0). Then we detected *hsa_circ_0000007* with RT-PCR and found that the expression of *hsa_circ_0000007* was significantly lower in cholesteatoma than normal skin. As mentioned above, circRNAs may regulate miRNAs and act as microRNA sponges. Therefore, we found *miR-508-3p* that was the targeted miRNA downstream of *hsa_circ_0000007* by using biological prediction software. Subsequently, we observed by RT-PCR that the expression of *miR-508-3p* in cholesteatoma was significantly higher than that in normal skin, and there was a statistically negative correlation with *hsa_circ_0000007*. Our experiment also demonstrated that the changes of *miR-508-3p* expression could affect the phenotypes of proliferation and apoptosis in cholesteatoma cells. In this study, cells with* miR-508-3p* NC, high* miR-508-3p* expression and low* miR-508-3p* expression were successfully obtained. The levels of Bax and Bcl-2 in cells of each group were determined by the ELISA kit. Bax and Bcl-2 are pro-apoptotic and anti-apoptotic proteins respectively, which play important roles in apoptosis 46. The experimental results revealed that compared with *miR-508-3p* NC group, *miR-508-3p* inhibitor can overtly elevate the level of pro-apoptotic factor Bax and lower the level of anti-apoptotic factor Bcl-2. On the contrary, *miR-508-3p* mimic can raise the level of anti-apoptotic factor Bcl-2 and lower the level of pro-apoptotic factor Bax. Then, EdU and TUNEL staining methods were adopted to detect the effect of *miR-508-3p* on the proliferation and apoptosis of cholesteatoma cells, respectively. The results suggest that *miR-508-3p* mimic can significantly promote the proliferation of middle ear cholesteatoma cells and suppress their apoptosis.

*MiR-508-3p* has also been reported in other diseases, and its biological functions are related to proliferation, apoptosis and invasion. For instance, Lin C et al. 47 find that overexpressing *miR-508* promotes, while silencing *miR-508* impairs, the aggressive phenotype of oesophageal squamous cell carcinoma both in vitro and in vivo. Another study demonstrated the functional role of *miR-508-3p* in promoting the proliferation, invasion and migration of ESCC cells. They also identified a *PCAT-1/miR-508-3p*/ANXA10 axis in mediating the promoting role of *miR-508-3p* as a potential therapeutic target of ESCC 48. But its mechanism of action in cholesteatoma has not been clarified.

We found a targeting relationship between *miR-508-3p* and *PTEN* through the TargetScan database analysis. Some CLIP-seq experiments also verified the targeted regulatory relationship between *PTEN* and *miR-508-3p* 49,50. In this study, we verified the targeting relationship between *miR-508-3p* and *PTEN* by Luciferase Reporter Assay. In addition, the expression of* PTEN* decreased significantly in *miR-508-3p* mimic group and increased significantly in *miR-508-3p* inhibitor group. This proves the targeted regulation of *miR-508-3p* on* PTEN* at the transcriptional level further. Moreover, the rescue experiment also proved that *PTEN* could reverse the proliferation trend of *miR-508-3p* mimic group cells. Therefore, we infer that *miR-508-3p* has an effect on the phenotype of cholesteatoma through *PTEN*.

PTEN (phosphatase and tens in homolog deleted on chromosome 10) (also named MMAC1/TEP1) was discovered in 1997 independently by three laboratories as a tumor suppressor of which the expression is often lost in tumors 51. Later studies established that PTEN is a negative regulator of a major cell growth and survival signaling pathway, namely the phosphatidylinositol-3-kinase (PI3K)/AKT signaling pathway 52. Phosphatidylinositol‑4,5‑bisphosphate 3‑kinase (PI3K) is activated and leads to protein kinase B (Akt) phosphorylated with the help of phosphoinositide‑dependent kinase, in the PI3K/Akt signal transduction pathway 53. Activated Akt may regulate multiple biological processes, including cell survival, metabolism, cell proliferation and growth, by affecting its downstream substrates 21. In order to investigate the regulatory mechanism of *miR-508-3p* in cholesteatoma, the protein expression levels of PTEN, PI3K and p-Akt in cholesteatoma cells were detected via Western Blotting in this study. As results presented in Fig. 6, compared with *miR-508-3p* NC group, *miR-508-3p* inhibitor can enhance PTEN protein level and impede PI3K and Akt protein expressions. On the contrary, *miR-508-3p* mimic can decrease the expression of PTN and increase the expression of PI3K and p-AKT.

To sum up, we concluded that *miR-508-3p* played a key role in the formation of cholesteatoma by regulating the PTEN/PI3K/Akt signaling pathway. While the overexpression of *miR-508-3p* in cholesteatoma is probably mediated by the regulation of upstream *hsa_circ_0000007*.
